# Design considerations for photoinitiator selection in cell-laden gelatin methacryloyl hydrogels†

**DOI:** 10.1039/d5bm00550g

**Published:** 2025-07-15

**Authors:** Elvan Dogan, Ann Austin, Ayda Pourmostafa, Swaprakash Yogeshwaran, Hossein Goodarzi Hosseinabadi, Amir K. Miri

**Affiliations:** a Department of Biomedical Engineering, Newark College of Engineering, New Jersey Institute of Technology 323 Dr Martin Luther King Jr Blvd Fenster Hall 624 (BME) Newark NJ 07102 USA am3296@njit.edu; b Department of Biomedical Engineering, Technical University of Eindhoven STO 4.37 Het Kranenveld 8 Bio-medical Engineering (BmE) department Eindhoven 5612 AZ The Netherlands h.goodarzi.hosseinabadi@tue.nl; c Department of Mechanical and Industrial Engineering, Newark College of Engineering, New Jersey Institute of Technology Newark NJ 07102 USA

## Abstract

Light-assisted bioprinting of protein-derived hydrogels has been widely used for tissue engineering and regenerative medicine. The practical challenges of the photoinitiators (PIs) are often overlooked in using photo-crosslinkable bioinks for *in situ* and *in vitro* applications. A higher concentration of PI is believed to increase the network density of a hydrogel thus reducing its mass transfer capacity, but PI can form reactive oxygen species (ROS) and cause unwanted side reactions around biological compartments. This study systematically investigates the role of ROS generation on mesenchymal stem cells encapsulated in gelatin-methacryloyl hydrogels when using type I PIs—*e.g.* lithium phenyl(2,4,6-trimethyl-benzoyl)phosphinate and 2-hydroxy-1-(4-hydroxyethyl-phenyl)-2-methyl-1-propanone, and type II PI—*e.g.* Eosin Y. The results reveal that higher concentrations of type I PIs provide a higher elastic modulus at the expense of enhanced ROS generation and a proportional decrease in viability. We report a novel hydrogel system with minimal PI loading where a reduction in elastic modulus is accompanied by a simultaneous decrease in pore size and ROS level leading to a significant increase in stem cell viability over one week of *in vitro* culture. In contrast, the type II PI reveals a moderate fluctuation of elastic modulus over a range of PI concentration correlated to fluctuations in ROS generation. Monitoring ROS level variations enables evaluation of each PI's impact on cell response, providing a strategy for the biofabrication of cell-laden constructs. This framework can inform the rational design of photo-crosslinkable hydrogels for light-assisted bioprinting and *in situ* crosslinking applications in regenerative medicine.

## Abstract 2

Light-assisted bioprinting of protein-derived hydrogels has been widely used for tissue engineering and regenerative medicine.

## Graphical abstract

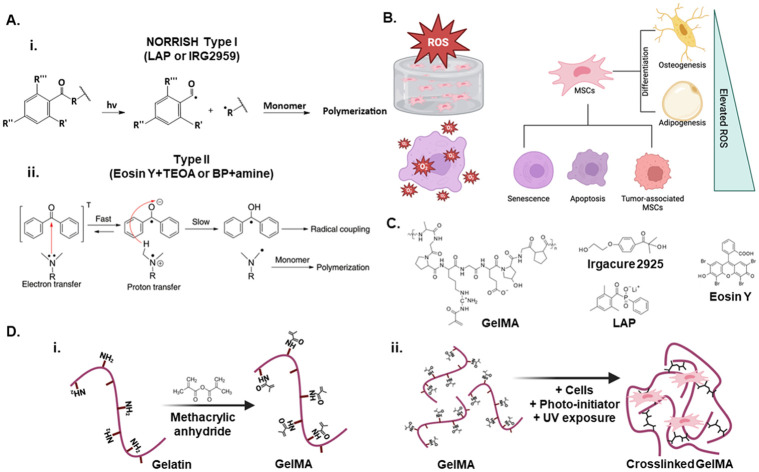

## 1. Introduction

Light-assisted biofabrication methods benefit from high-resolution and rapid creation of photo-crosslinkable bioinks as a functional microtissue or scaffold model.^1,2^ An optimum bioink benefits from high cell viability,^3^ tuning extracellular matrix (ECM),^4,5^ and functional cell markers.^6^ The ability to finely control the crosslinking process within hydrogel-based bioinks allows tailoring of the biophysical properties of the final construct.^2,7^ A key component is the photoinitiator (PI), which initiates photopolymerization, which can impact cell response, such as oxidative damage, reduced proliferation rates, and altered mechanical properties.^8,9^ The role of PI in regulating cell behavior in bioprinted constructs has been overlooked in many published works.^10^ For example, a byproduct of PI reactions is the reactive oxygen species (ROS) that contain oxygen when the PI absorbs the excitation light energy. The ROS signal represents a group of oxygen-containing entities (*i.e.*, atoms, ions, molecules, or radicals), including singlet oxygen (^1^O_2_), superoxide anion (O_2_^−^), hydroxyl radical (OH˙), and hydrogen peroxide (H_2_O_2_). They are pivotal in cellular signaling, wound healing, and tissue regeneration. The overproduction of ROS can lead to oxidative stress, which can damage embedded cells.^11^ This dual nature of ROS highlights the need for its delicate balance to maintain viability, cell fate, and functionality within the constructs.

Several factors must be considered for the PI design challenges in light-assisted bioprinting. First, crosslinking density and cell compatibility should be balanced, as some PIs can excessively generate ROS that compromise cell viability and micro-tissue function.^12^ Second, the PI must be carefully matched with the hardware (*i.e.*, light source wavelength) to achieve optimal resolution and polymerization kinetics with minimal ROS generation.^2^ Third, the scattering and absorption behavior of light in the bioink affects the fidelity and mechanics of the final construct.^2,12^ Light penetration and scattering variability can impede crosslinking, which leads to structural defects. Fourth, excessive crosslinking density can decrease the degradation rate and pore size, impairing construct biodegradation and cell nutrition. The PI should maintain the ROS at a safe range, ensure uniform curing at clinically relevant resolution, and allow control over the mechanical properties and degradation rate of bioprinted constructs.

Type I PIs produce reactive intermediates to initiate polymerization from the excited triplet state. Type II PIs can abstract hydrogen from the donors and undergo a photoinduced electron transfer and fragmentation process to make reactive intermediates. The reactive intermediates may be radicals or cations that initiate radical or cationic photopolymerization. These free radicals react with the monomer and form radicalized monomers, which further react with other monomer units to form a polymer chain.^2,12^ Type I PIs, including lithium phenyl(2,4,6-trimethyl-benzoyl)phosphinate (LAP) and 2-hydroxy-1-(4-(hydroxymethyl)-phenyl)-2-methyl-1-propanone (Irgacure 2959, I2959), directly produce free radicals upon light absorption by undergoing cleavage.^13^ In contrast, a type II PI such as Eosin Y requires a co-initiator such as triethanolamine (TEOA) to abstract hydrogen, which is necessary for free radical formation.^13^ The radical generation rate depends on PI concentration, light intensity, quantum yield, and absorbance fraction (modeled *via* Beer–Lambert law as shown in eqn (3) of ESI†).

**Fig. 1:**
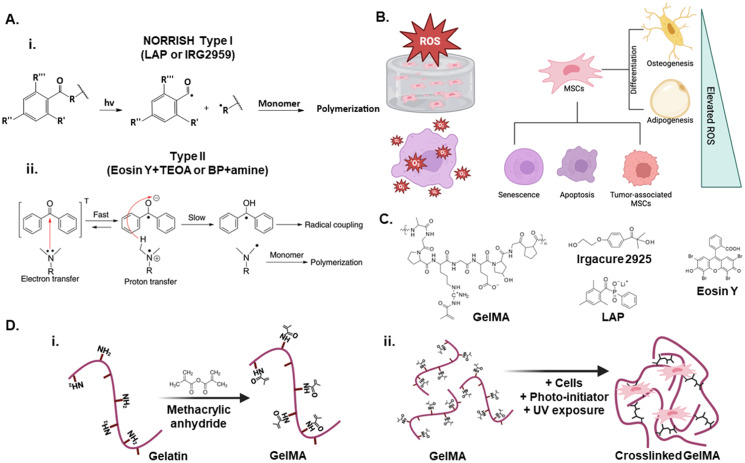
Photoinitiator (PI)-mediated crosslinking mechanisms and biological impact in gelatin methacryloyl (GelMA) hydrogels. (A) Schematic of GelMA synthesis and distinct crosslinking pathways: (i) type I PIs (LAP or Irgacure 2959) initiate crosslinking *via* Norrish type I cleavage and direct radical generation upon UV (365 nm) exposure; (ii) type II PIs (Eosin Y + TEOA or benzophenone + amine) initiates crosslinking *via* triplet energy transfer or photoinduced electron transfer under visible light (500–590 nm), generating radicals indirectly (adapted by Ghosh *et al.*^15^). (B) Elevated reactive oxygen species during the photoinitiation process can affect mesenchymal stem cells in GelMA hydrogel, leading to senescence, apoptosis, and formation of tumor-associated mesenchymal stem cells. These changes can subsequently alter differentiation pathways, including osteogenesis (bone formation) and adipogenesis (fat formation). (C) Chemical structure of a GelMA macromer is shown beside that of commonly used PIs in GelMA crosslinking. (D) Upon exposure to UV light in the presence of a PI, GelMA undergoes photo-polymerization to form a crosslinked hydrogel. (i) Gelatin is chemically modified with methacrylic anhydride to produce Gelatin Methacryloyl (GelMA). (ii) Chemical structure of a GelMA macromer is shown beside that of commonly used PIs in GelMA crosslinking.

Among common bioinks, gelatin methacrylate (GelMA) is a widely-used photo-responsive biomaterial derived from gelatin—denaturalized from collagen protein, with advantages such as good cell adhesion, proliferation, and differentiation.^14^ Upon light-based crosslinking, its methacryloyl group allows fine-tuning of the mechanical properties and facilitates the creation of a highly structured and cell-supportive environment to mimic the ECM.^15^ In this work, we used GelMA, as a backbone, along with three PIs (LAP, I2959, and Eosin Y) to assess their impact on the physical, morphological, mechanical, and biological properties of the constructs (see Fig. 1). Stem cells were used for biological assessment of the PIs. The ROS levels were quantified at predetermined intervals (1, 24, and 48 hours). Mechanical properties of the hydrogels were also evaluated to investigate the correlation of the ECM's elastic modulus, ROS generation, and cellular response. The impact of PI type and concentration on hydrogel elasticity, cellular adhesion, viability, and ROS generation can be used for optimizing bioprinting processes and improving biocompatibility.

## 2. Materials and methods

### 2.1 Cell preparation

Bone-marrow-derived human mesenchymal stem cells (MSCs, PT-2501, Lonza, Walkersville, MD) were cultured in α-minimum essential medium (αMEM; Corning, NY, USA) supplemented with 10% fetal bovine serum (FBS), 2 mM l-glutamine (Gibco), and 1% penicillin–streptomycin (Gibco) at 37 °C and 5% CO_2_. The medium was changed every two days, and the experiments used the third passage. Cells were suspended after trypsinization using 1 mL of trypsin–EDTA solution (Corning, Manassas, VA) in a 15 mL centrifuge tube and centrifuged (Eppendorf, Centrifuge 5810 R, Hamburg, Germany) for 5 min at 350*g* for hydrogel encapsulation.

### 2.2 GelMA synthesis and precursor preparation

GelMA was synthesized according to our established protocol.^7^ In a glass flask, 100 mL of Dulbecco's phosphate-buffered saline (DPBS) (Sigma-Aldrich, St Louis, MO, USA) and 10% w/v porcine skin gelatin (CAS 9000-70-8; Sigma-Aldrich, St Louis, MO, USA) were mixed. The flask was covered to prevent evaporation and was magnet stirred for an hour at 60 °C until fully dissolved. 5 ml methacrylic anhydride was slowly pipetted into the solution. The temperature was turned down to 50 °C and the solution was allowed to stir and react for an hour. 5× pre-warmed DPBS was added to the solution to stop the reaction. Dialysis tubes (12–14 kDa cut-off) were then submerged in deionized (DI) water for a week at 40 °C. DI water was changed twice a day for the first few days. After a week, the solution from the dialysis tube was transferred into centrifuge tubes, stored at −80 °C, and once fully frozen, freeze-dried for one week until the synthesized GelMA demonstrated a white foam structure.

GelMA precursor solution was prepared (14% w/v) with DPBS pre-warmed at 50 °C. The solution was diluted to a final concentration of 5% w/v GelMA with various concentrations (0.3 to 1% w/v) of I2959 (CIBA Chemicals). Similarly, 5% w/v GelMA solution with multiple concentrations of LAP was prepared: 0.01 (low) to 0.1% w/v (high). To prepare 5% (w/v) GelMA with different concentrations of Eosin Y (Sigma Aldrich, St Louis, MO, USA; CAS: 17372-87-1), the following Eosin Y concentrations were used: 0.05 nM (low), 0.075 nM, 0.1 nM (medium), 0.25 nM, and 0.5 nM (high). Triethanolamine (TEOA) was added to each solution at a final concentration of 1 μM, and *N*-vinylpyrrolidone (NVP) was included at a concentration of 0.05 mL to enhance the polymerization process. All hydrogel precursor solutions were filtered through 0.2 μm filters, cylindrical hydrogels for compression, swelling, and degradation tests were made using PDMS molds after crosslinking at 365 nm light and 10 mW cm^−2^ intensity for threshold time in I2959 samples (3 min) and in LAP samples (2.5 min) for the lowest concentration and consistency among our groups (see Fig. S1†). All samples were conditioned in an incubator at 37 °C for 24 hours to reach equilibrium prior to mechanical characterization.^16^

Threshold crosslinking times were determined for Eosin Y by increasing crosslinking time gradually and conducting compression test. Crosslinking times for each group were selected based on the first statistical significance observed in compressive modulus, indicating threshold crosslinking occurred (Fig. S1†).

### 2.3 Mechanical characterization

Standard compression testing was performed to determine the stiffness of the material under physical load. We followed a standard protocol^17^ and fabricated disk-shape samples (10 × 5 mm, diameter × height). GelMA hydrogel samples with various concentration of LAP and I2959 were crosslinked under 365 nm UV light using PDMS mold. We analyzed the elastic modulus of 3 different hydrogel sets (*n* = 3) for each group to evaluate the concentration effect of PI on hydrogel stiffness properties while keeping MA degree the same. Then, samples placed between the metal flat plates of the universal testing machine (Instron, MA, USA) and compression tests were performed using 1 mm min^−1^ strain rate. The cross-sectional area of each hydrogel was calculated as the ratio of weight to height. The Young's modulus was calculated as the slope of the stress–strain curve at 10% engineering strain.

### 2.4 Degradation test

To measure the degradation ratio of the hydrogels, three samples from each group (10 × 5 mm, diameter × height) were crosslinked and placed in 2 mL of collagenase solution (3818971 Collagenase I, Sigma Aldrich, USA) at 2.5 U mg^−1^ in a 24-well plate. The samples were incubated at 37 °C. Every hour, the samples were removed and weighed. The degradation ratio was calculated using the following formula: (*W*(*t*) − *W*(0))/*W*(0) × 100, where *W*(0) is the initial weight of the sample before immersion in deionized water, and *W*(*t*) is the weight of the sample at a given time point.

### 2.5 Swelling test

To measure the equilibrium swelling of the hydrogels, we photocrosslinked the GelMA into disk-shaped hydrogels (10 × 5 mm, diameter × height; 3 samples per group) in a PDMS mold. The samples were then freeze-dried, and their dry mass (*W*_d_) was measured. Next, the hydrogels were rehydrated with 2 ml of DPBS buffer at 37 °C, and the swollen weights (*W*_s_) were recorded up to day 5. The percentage degree of swelling (%) was measured as [(*W*_s_ − *W*_d_)/*W*_d_] × 100, where *W*_s_ is the weight of swollen GelMA hydrogel at measuring time, and *W*_d_ is the dry weight of each GelMA hydrogel after freeze-drying.

### 2.6 Scanning electron microscopy

The structure of the GelMA hydrogel with varying type and concentration of PI was analyzed using a scanning electron microscope (SEM). Hydrogels with lowest, middle and highest concentration of PI (LAP, I2959, and Eosin Y) were placed in incubator in DPBS for 24 h at 37 °C and swollen hydrogels were frozen (−80 °C) and were subsequently lyophilized (*n* = 3) for each group. The lyophilized samples were cut and their cross-sections were coated with Au–Pd using a turbo sputter coater (EMITECH, K575X). SEM images were acquired (JSM-7900F Schottky Field Emission Scanning Electron Microscope) at a voltage of 5 kV. The quantifications of the porosity and aspect ratio were analyzed by ImageJ software (see Fig. S2†).

### 2.7 Bioink preparation

For hydrogel preparation, 1.5 × 10^3^ MSCs were encapsulated in 50 μl of 5% w/v GelMA (*n* = 3) in a 96 well plate and UV crosslinked under 365 nm 10 mW cm^−2^ intensity. 500 μl of MSC media was added throughout the experiment.

### 2.8 Cell assays for viability evaluation and cell proliferation

The viability of MSCs was assessed using the CCK-8 assay (96992 cell counting kit-8, Sigma Aldrich) and live/dead cell staining. GelMA solutions (50 μL each) containing MSCs (15 × 10^3^ cells) were injected into 96-well plates and polymerized under UV light for 2.5 minutes for LAP and 3 minutes for I2959. The hydrogels were then covered with 200 μL of MSC culture medium, and the culture was maintained up to day 7. At each time point, MSCs were incubated in medium containing 10% CCK-8 reagent for 2 hours under standard culture conditions. The medium's optical density (OD) was measured at 450 nm using a microplate reader. For live/dead cell staining, MSCs were washed with PBS and stained with Calcein AM/PI (BioLegend, San Diego, CA, USA) at 37 °C for 1 hour, following the manufacturer's protocol at day 0 and 1. Fluorescence microscopy was used to capture images, where live cells stained with calcein AM emitted green fluorescence, and dead cells stained with PI emitted red fluorescence. Each image was then analyzed by ImageJ.

### 2.9 Cellular ROS detection assay

ROS level was determined for MSC cells with the standard ROS detection kit (ab113851; Abcam, USA) using varying types and concentrations of PI. The assay's buffer and DCFDA solution were prepared following the manufacturer's protocol. MSCs were encapsulated in 5% GelMA containing different concentrations of LAP, I2959, and Eosin Y (*n* = 3) by photocrosslinking. Post-crosslinking, the MSC-laden GelMA constructs were placed in 200 μl of MSC media. After one hour, the media was removed and the constructs were washed twice with 1× buffer solution followed by adding the DCFDA solution. After a 45-minute incubation, the DCFDA solution was removed, and the constructs were washed twice with 1× buffer. Control was set without using ROS assay (negative control) as well as using TBHP (positive control) solution. ROS generation was quantified using a plate reader at 485/535 nm wavelength.

### 2.10 Focal adhesion staining

This staining was performed to determine F-actin alignment and cell morphology using an actin cytoskeleton and focal adhesion staining kit (FAK100; Millipore, Bedford, MA). MSC cells were encapsulated in GelMA hydrogels with varying LAP and I2959 concentrations up to day 5 and then fixed with 4% paraformaldehyde. Hydrogels were washed with 1× wash buffer and permeabilized with 0.1% Triton X-100 in 1× PBS. A blocking solution was applied at room temperature. Cell adhesion morphology was visualized with F-actin and vinculin staining. The primary antibody, anti-vinculin, was diluted in a blocking solution (1 : 200) and incubated with the hydrogels. After washing with a 1× wash buffer, hydrogels were incubated with goat anti-mouse FITC-conjugated secondary antibodies diluted in 1× PBS, along with TRITC-conjugated phalloidin (1 : 200). For nuclear counterstaining, hydrogels were incubated with DAPI (1 : 1000) followed by washing.

### 2.11 Statistical analysis

Statistical analysis was performed in IBM SPSS (NY, USA) statistical tool. A one-way analysis of variance (one-way ANOVA with Tukey's *post hoc*) test and two-tailed *T*-tests were used for data analysis. A value of *p* < 0.05 was considered to be statistically significant.

## 3. Results and analysis

### 3.1 Swelling and degradation behavior of GelMA hydrogels

#### LAP-crosslinked GelMA

**Fig. 2:**
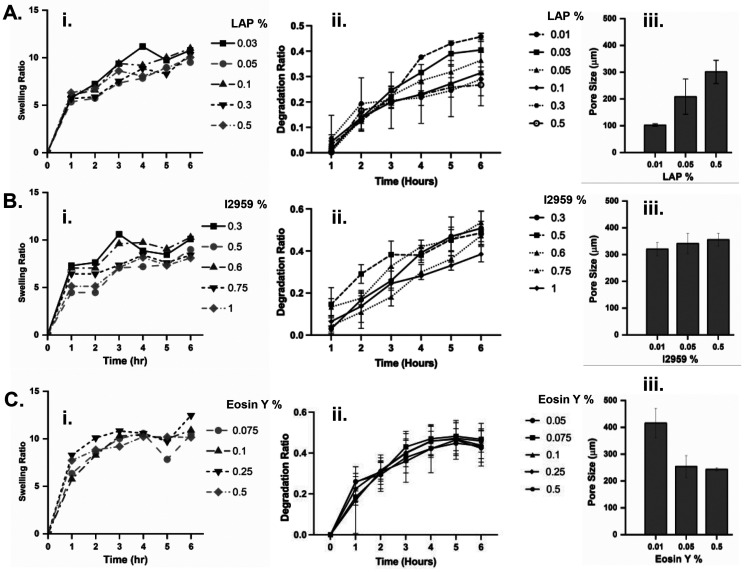
Characterization of GelMA hydrogels made of three different PIs: A. LAP, B. I2959, C. Eosin Y. i. Swelling ratio for different PI concentrations. ii. The enzymatic degradation ratio for different PI concentrations in w/v %. iii. Pore size analysis was obtained from SEM micrographs of the crosslinked GelMA hydrogels at the relevant PI concentrations. *n* = 3.

The swelling ratio of GelMA hydrogels in Fig. 2A-i remained relatively stable across different LAP concentrations. The sample with the lowest LAP concentration exhibited the highest swelling ratio, which match with SEM image analysis (Fig. 2A-iii and Fig. S2†) showing that at lower LAP concentrations the spongy sample reveals more homogenous distribution of small pores with thinner walls, contributing to the increased swelling ratio. The enzymatic degradation profile of LAP in GelMA hydrogels was assessed over six hours (Fig. 2A-ii). A time-dependent increase in degradation was observed for all LAP concentrations. Hydrogels with higher LAP concentrations exhibited a slower degradation rate, supporting the notion that higher LAP contributes to forming a denser crosslinked hydrogel network with lower degradation kinetics.

#### I2959-crosslinked GelMA

The swelling ratio of GelMA hydrogels in Fig. 2B-i indicates that the swelling ratio fluctuated slightly across different concentrations, with all samples exhibiting moderate changes over time. Hydrogels crosslinked with higher concentrations of I2959 showed a decreased swelling ratio compared to those with lower concentrations (0.3% and 0.5% w/v) at day 5. The enzymatic degradation of I2959 in GelMA was assessed over six hours (Fig. 2B-ii). A concentration-dependent decrease in degradation was observed, where hydrogels crosslinked with lower concentrations of I2959 showed more rapid degradation compared to those with higher concentrations. This pattern highlights the proportional influence of I2959 concentration on the stability of the hydrogel matrix against degradation. The pore size remained similar for all mass concentrations, showing a consistent performance of I2959 compared to other PIs.

Our swelling ratio trends for type I PIs are consistent with prior reports that increased crosslink density reduces equilibrium swelling.^8,17^ Degradation profiles align with prior studies showing that higher crosslink density slows enzymatic degradation in GelMA.^18,19^ This is because increased crosslinked side chain density makes the network more resistant to enzymatic cleavage by enzymes such as collagenase. Greater photopolymerization results in a denser network, which requires more enzymatic activity and longer time to degrade.

#### Eosin Y-crosslinked GelMA

Swelling and degradation profiles exhibited trends independent of Eosin Y concentration (Fig. 2C-i and ii). Unlike type I PIs, in this type II PI a concentration-dependent increase in swelling was not observed. It is speculated that an increase in Eosin Y concentration does not lead to enhanced crosslinking within the hydrogel network; rather, higher levels of Eosin Y may adversely affect crosslinking by inhibiting the formation of covalent bonds between methacrylated groups in GelMA. Similarly, degradation showed statistically non-significant mass loss across different Eosin Y concentrations. Interestingly, an increased concentration of Eosin Y proportionally leads to formation of significantly smaller pores in GelMA (Fig. 2C-iii and Fig. S2†). This reduction behavior can be used to control the delivery mechanisms using this type of PI.

The more complex swelling behavior observed with Eosin Y is consistent with the unique indirect radical formation mechanism of type II PIs, in agreement with reports by Noshadi *et al.*^20^ This complexity arises from the involvement of a co-initiator (TEOA) and a polymerization accelerator (NVP, *N*-vinylpyrrolidone) in the system. Optimizing the concentrations of both the co-initiator and accelerator becomes a critical factor. Moreover, the reaction kinetics between the co-initiator, accelerator, and PI require further investigation to fully elucidate the complex mechanism of photoinitiation in Eosin Y-based systems.

It is notable that SEM imaging of freeze-dried hydrogels shows the apparent porosity, involving structural changes caused by lyophilization.^21^ This process can lead to collapse or distortion of the native pore architecture, and thus may not accurately represent the hydrated state, as previously reported in the literature.^20,22,23^ All groups were processed under identical conditions, and the SEM-based pore size analysis serves as a valid comparative method to evaluate relative differences in microstructure among experimental groups. This approach is consistent with previous studies we published employing similar methodologies for structural comparison.^7,19^

### 3.2. Mechanical properties of GelMA hydrogels and MSC viability

#### LAP-crosslinked GelMA

**Fig. 3:**
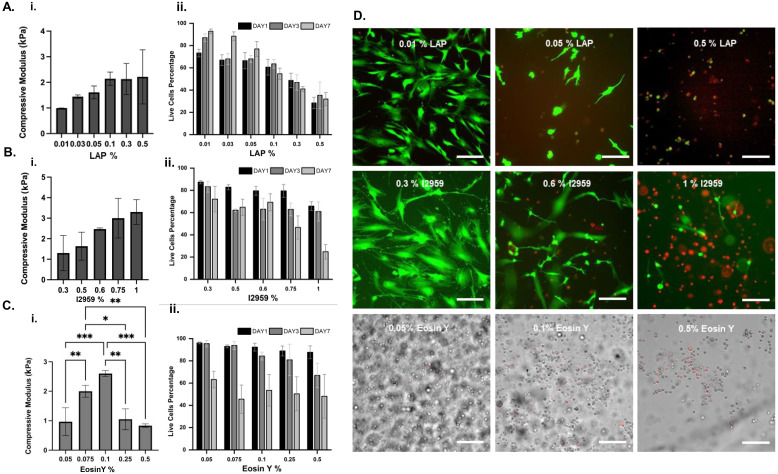
Characterization of A. LAP, B. I2959, C. Eosin Y in gelatin-based systems. i. Compressive modulus (kPa), ii. Cell viability (%), D. Representative live–dead images for several concentrations of photo-initiators in GelMA 5% obtained at day 7 after culture. For LAP and I2959, live cells are shown in green and dead cells are shown in red. For Eosin Y, the bright field images are shown in gray and dead cells are shown in red (see ESI, Fig. S3–S5†). Scale bars represent 100 μm. *n* = 3.

**Fig. 4:**
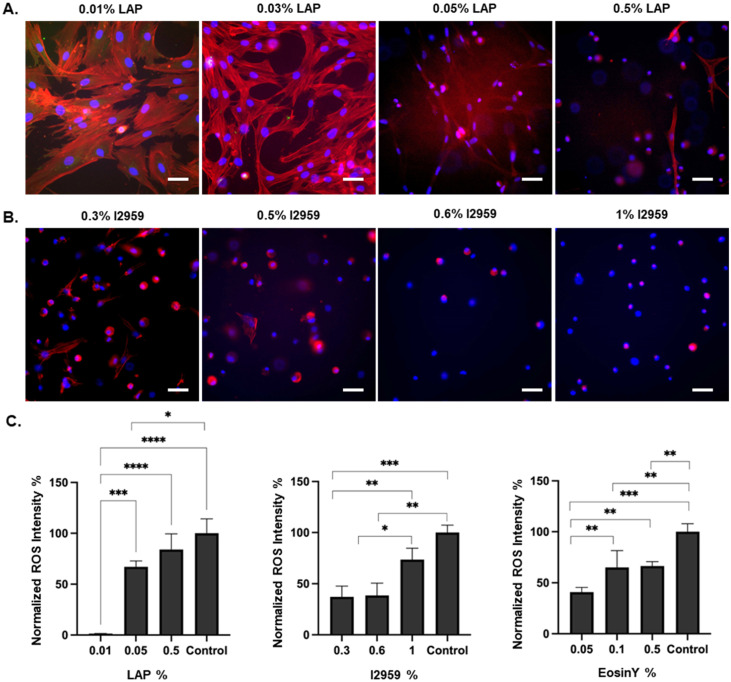
Focal adhesion staining images (red: actin, blue: DAPI) for A. LAP, B. I2959, in the gelatin-based system, C. normalized intensities of ROS generation in hydrogels prepared with different concentrations of LAP, I2959, and Eosin Y. Scale bars represent 100 μm. *n* = 3.

The elastic modulus of hydrogels crosslinked with various concentrations of LAP was measured to assess the impact of LAP concentration on mechanical properties (Fig. 3A-i). The compressive modulus increased with LAP concentration, indicating that higher LAP concentrations resulted in stiffer hydrogels. This trend suggests that higher LAP concentrations effectively crosslink the GelMA network, enhancing its mechanical strength. These results are consistent with our previous findings^7,24^ using similar GelMA bioinks and confirm that a higher LAP concentration proportionally contributes to increasing the crosslink density in the hydrogel network, decreasing the degradation rate and enhancing the elastic modulus of the network. At LAP concentrations over 0.1% w/v, the viability of encapsulated MSCs decreases over the culture period (Fig. 3A-ii), which correlates to the elevated ROS levels in the system, as we will discuss later in Fig. 4C.

#### I2959-crosslinked GelMA

The compressive modulus of GelMA hydrogels was measured to evaluate the effect of I2959 concentration on mechanical strength (Fig. 3B-i). A clear trend of increasing compressive modulus was observed with higher I2959 concentrations, with the highest modulus recorded at 1% w/v. These results suggest that increased crosslinking density, achieved with higher I2959 concentrations, enhances the rigidity and mechanical integrity of the hydrogels. These results are consistent with our previous findings^7,24^ using similar GelMA bioinks and confirm that a higher I2959 concentration proportionally contributes to increasing the crosslink density and enhancing the elastic modulus of the network.^19^ Both ROS level generation and too high elastic modulus (*i.e.* >2.5 kPa at 0.75 and 1% concentrations) can adversely contribute to the decrease in MSC viability in these constructs (Fig. 3B-ii).

#### Eosin Y-crosslinked GelMA

Eosin Y—a type II PI activated by visible light (500 to 590 nm), was used to crosslink GelMA hydrogels at varying concentrations (Fig. 3C-i). The compressive modulus of the hydrogels increased significantly with increasing Eosin Y concentration from 0.05 to 0.1% followed by a sharp decrease at 0.25 and 0.5% (w/v) Eosin Y concentrations. Hydrogels crosslinked with 0.1% Eosin Y exhibited the highest compressive modulus, indicating presence of an optimal PI concentration to maximize the hydrogel elastic modulus. Higher Eosin Y concentrations led to decreased pore size hindering the nutrition and waste transition through the hydrogel network to retain the MSCs viability.

### 3.3. Cell adhesion, oxidative stress, and viability

#### LAP-crosslinked GelMA

Focal adhesion analysis clearly shows that increasing LAP concentrations proportionally leads to weaker cell attachments and cell spreading, as indicated in Fig. 4A. To assess the oxidative stress induced by LAP, the normalized intensity of ROS in LAP-treated cells was quantified (Fig. 4C). An increase in ROS intensity was observed with increasing LAP concentrations, particularly at 0.05% and 0.5% w/v, indicating that higher LAP levels may induce oxidative stress in cells. Cell viability that was evaluated through a live–dead assay (Fig. S2 and Fig. 3A-ii) showed that the percentage of live cells decreased with increasing LAP concentrations, with the most significant reduction observed at 0.5% w/v (Fig. 3D). These observations reveal a dose-dependent increase in ROS generation in I2959 samples, leading to reduced cell viability. The ESI also supported the dose-dependent cytotoxic effect of LAP (see Fig. S8†), where the LAP free radical generation rate is more susceptible to its dose dependency than I2959. This result explains the lower variation related to dose-dependent ROS generation in I2959 samples compared to LAP samples (see Fig. S6–S9†).

#### I2959-crosslinked GelMA

Cell viability in the hydrogels crosslinked with various concentrations of I2959 was assessed using a live–dead assay (Fig. S3† and Fig. 3B-ii). The percentage of live cells decreased progressively with increasing I2959 concentration. This trend was consistent across all time points (day 1, 3, and 7), with the most pronounced reduction in cell viability observed at 1% w/v. Focal adhesion analysis further confirmed that lower concentrations of I2959 (0.3% and 0.5% w/v) facilitated better cell attachment and spreading, as indicated by the fluorescent images (Fig. 4B). An increase in ROS intensity was observed with increasing I2959 concentrations, particularly at 1% w/v, suggesting that higher I2959 levels may induce oxidative stress in cells (Fig. 4B) compared to 0.3 and 0.6% w/v.

#### Eosin Y-crosslinked GelMA

The live–dead assay was employed to assess cell viability in GelMA crosslinked with various concentrations of Eosin Y (Fig. 3C-ii). The results demonstrate a concentration-dependent reduction in cell viability, with higher concentrations of Eosin Y (0.25% and 0.5% w/v) leading to more pronounced cell death. The observed cytotoxicity at higher concentrations could be attributed to the higher degree of crosslinking, which might impede nutrient and waste diffusion, adversely affecting cell survival. A slight increase in ROS intensity was observed with increasing Eosin Y concentration from 0.05% to 0.1% w/v while increasing the Eosin concentration above 0.1% w/v retained ROS level at a constant amount, indicating that higher Eosin Y concentrations may contribute to fixed oxidative stress for the cells (Fig. 4C). The reduced viability in these constructs can be attributed to smaller pore size and inefficient mass transport within the network structure.

## 4. Discussions and concluding remarks

The data analysis is discussed from three different angles to elucidate the role of three different PIs in the gelatin based hydrogels. First, we rated the PIs for their implementation at *in vitro* and *in vivo* models. Second, we considered the control over biofabrication speed and quality (*e.g.*, resolution). Third, we studied the role of the PI in regulating cell response for potential desired output by quantification of generation of the ROS level. We discuss these topics separately.

Our data showing ROS-dependent reduction in MSC viability with increasing PI concentration aligns with trends reported by previous literature.^9,25,26^ Our time-resolved ROS data (1 h, 24 h, 48 h) in cell-laden constructs add new insights beyond prior studies that primarily used endpoint or acellular measurements. We demonstrated focal adhesion disruption at higher ROS levels, complementing previous reports on oxidative stress effects on cytoskeletal integrity.^9^

Several earlier studies have analyzed the cytotoxicity and functional performance of photoinitiators in GelMA hydrogels, with each contributing information to a particular PI-cell interaction or polymerization efficiency. For instance, Nguyen *et al.*^27^ indirectly quantified ROS generation by LAP through a fluorescence-based NADH oxidation assay, where photogenerated ROS converted NADH (the reduced form of nicotinamide adenine dinucleotide) into NAD^+^ (*i.e.* oxidized form). The rate of fluorescence decay under UV-A (*i.e.* ultraviolet light with wavelengths between 320–400 nm) and visible light exposure was used as a proxy for ROS activity, allowing for comparative assessment of the photosensitizing potential of LAP. They investigated LAP-induced cytotoxicity in renal epithelial cells specifically regarding photosensitization pathways but did not investigate mechanical or ROS-associated responses in 3D constructs. Unlike the NADH oxidation assay, which measures ROS generation in acellular systems, the assay used in this study is DCFDA (*i.e.* 2′,7′-dichlorodihydrofluorescein diacetate as a fluorescent probe) which quantifies intracellular ROS levels, providing a more biologically relevant indicator of oxidative stress in cell-laden hydrogels. Pahoff *et al.*^28^ emphasized gelatin source and PI type dependence of chondrocyte redifferentiation but did not quantify ROS or simulate degradation kinetics. Xu *et al.*^29^ contrasted LAP and I2959 in vascular-like constructs and reported viability trends but did not incorporate time-resolved ROS measurements or material degradation modeling. Recently, Duymaz *et al.*^30^ investigated photoinitiation kinetics of GelMA networks systematically but did not investigate biological outcomes or integrate mechanical and microstructure analysis.

In contrast, our study for the first time combines biophysical, chemical, and biological assessments by (i) directly comparing three PIs—two type I and one type II—on identical GelMA platforms, (ii) analyzing ROS intensity at multiple time points and correlating these with stem cell viability and adhesion, and (iii) employing UV-Vis-based degradation kinetics to define free radical generation. These multimodal findings collectively present a mechanistic image of how PI type and concentration regulate cell fate and hydrogel performance, which is vital for the rational design of photocrosslinked systems in regenerative applications.

First, the role of different PIs in light-based bioinks has been overlooked as a governing component. Here, we showed how the PI can change the biophysical and microstructural properties of the bioprinted constructs. The mass concentration of PI and the exposure timing to the relevant light intensity can be well designed to control the elastic properties and porosity in all three cases. However Eosin Y showed a higher control over the pore size of bioprinted constructs while their elastic properties can be better tuned with LAP and I2959. The swelling and degradation behavior are well similar for the three PIs and the type of PI has a limited role for such hydrogel features.

Second, the PI selection is to optimize the biofabrication quality. This can be done by increasing the concentration of type I PIs, which leads to a proportional increase in the hydrogel's elastic modulus at the cost of significantly elevated ROS levels and oxidative stress, reducing MSC viability. In contrast, the type II PI reached its maximum elastic modulus at an optimal concentration, which was correlated to the level of ROS generation. This results in more stable cell viability across a range of type II PI concentrations. Monitoring ROS fluctuations offers a helpful method to assess the impact of each PI on cell behavior, providing an effective strategy for the biofabrication of cell-laden constructs.

Third, we showed the level of cell response by a standard kit for quantification of ROS level and conventional metabolic activity markers. This cell response becomes more critical and impact the host cells when we apply the PI for *in vivo* applications and *in situ* delivery of biomaterials.

## Conflicts of interest

The authors declare that there are no conflicts of interest.

## Supplementary Material

BM-014-D5BM00550G-s001

## Data Availability

The data supporting this article have been included as part of the ESI.†
