# Correction: Design considerations for photoinitiator selection in cell-laden gelatin methacryloyl hydrogels

**DOI:** 10.1039/d5bm90068a

**Published:** 2025-10-27

**Authors:** Elvan Dogan, Ann Austin, Ayda Pourmostafa, Swaprakash Yogeshwaran, Hossein Goodarzi Hosseinabadi, Amir K. Miri

**Affiliations:** a Department of Biomedical Engineering, Newark College of Engineering, New Jersey Institute of Technology 323 Dr Martin Luther King Jr Blvd Fenster Hall 624 (BME) Newark NJ 07102 USA am3296@njit.edu; b Department of Biomedical Engineering, Technical University of Eindhoven, STO 4.37, Het Kranenveld 8, Bio-Medical Engineering (BmE) Department Eindhoven 5612 AZ The Netherlands h.goodarzi.hosseinabadi@tue.nl; c Department of Mechanical and Industrial Engineering, Newark College of Engineering, New Jersey Institute of Technology Newark NJ 07102 USA

## Abstract

Correction for ‘Design considerations for photoinitiator selection in cell-laden gelatin methacryloyl hydrogels’ by Elvan Dogan *et al.*, *Biomater. Sci.*, 2025, https://doi.org/10.1039/d5bm00550g.

The authors regret that there were errors in the units in the sentences in lines 40–48 in left column on page 3, lines 42–45 in the right column on page 3, lines 41–44 in the right column on page 4, lines 12–19 in the right column on page 6 and lines 3–15 in the right column on page 8 of the original article. These sentences should read as follows:

“2.2 GelMA synthesis and precursor preparation” – To prepare 5% (w/v) GelMA with different concentrations of Eosin Y (Sigma Aldrich, St Louis, MO, USA; CAS: 17372-87-1), the following Eosin Y concentrations were used: 0.05 mM (low), 0.075 mM, 0.1 mM (medium), 0.25 mM, and 0.5 mM (high). Triethanolamine (TEOA) was added to each solution at a final concentration of 1 mM, and *N*-vinylpyrrolidone (NVP) was included at a concentration of 95 mM to enhance the polymerization process.

“2.5 Swelling test” – The samples were then freeze-dried, and their dry mass (*W*_d_) was measured. Next, the hydrogels were rehydrated with 2 ml of DPBS buffer at 37 °C, and the swollen weights (*W*_s_) were recorded up to 6 hours.

“3.1: I2959-crosslinked GelMA” – Hydrogels crosslinked with higher concentrations of I2959 showed a decreased swelling ratio compared to those with lower concentrations (0.3% and 0.5% w/v) at 6 hours.

“3.2: Eosin Y-crosslinked GelMA” – The compressive modulus of the hydrogels increased significantly with increasing Eosin Y concentration from 0.05 to 0.1 mM followed by a sharp decrease at 0.25 and 0.5 mM Eosin Y concentrations. Hydrogels crosslinked with 0.1 mM Eosin Y exhibited the highest compressive modulus, indicating presence of an optimal PI concentration to maximize the hydrogel elastic modulus.

“3.3: Eosin Y-crosslinked GelMA” – The results demonstrate a concentration-dependent reduction in cell viability, with higher concentrations of Eosin Y (0.25 and 0.5 mM) leading to more pronounced cell death. The observed cytotoxicity at higher concentrations could be attributed to the higher degree of crosslinking, which might impede nutrient and waste diffusion, adversely affecting cell survival. A slight increase in ROS intensity was observed with increasing Eosin Y concentration from 0.05 to 0.1 mM while increasing the Eosin concentration above 0.1 mM retained ROS level at a constant amount, indicating that higher Eosin Y concentrations may contribute to fixed oxidative stress for the cells (Fig. 4C).

The authors regret labelling error in Fig. 2, 3, 4, S1, S2 and S5 in the original manuscript. The correct versions of Fig. 2, 3, 4, S1, S2 and S5 is as shown below.

**Fig. 2:**
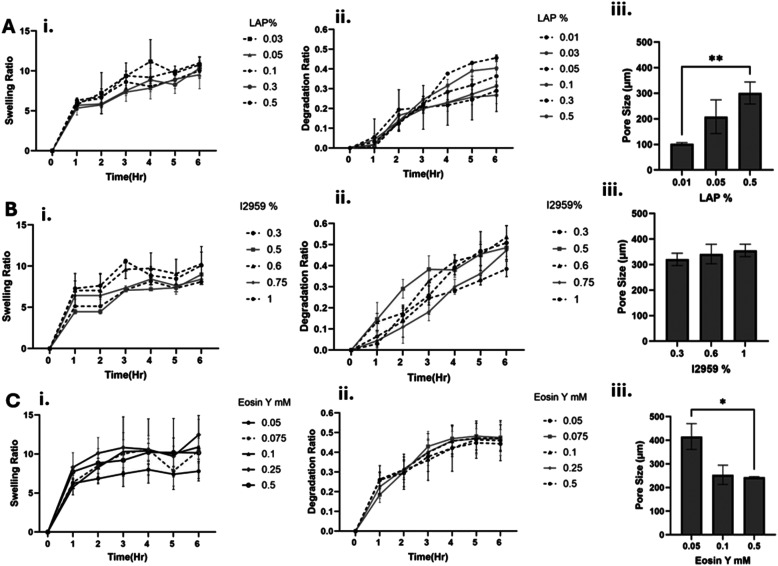
Characterization of GelMA hydrogels made of three different PIs: A. LAP, B. I2959, C. Eosin Y. i. Swelling ratio for different PI concentrations. ii. The enzymatic degradation ratio for different PI concentrations in w/v %. iii. Pore size analysis was obtained from SEM micrographs of the crosslinked GelMA hydrogels at the relevant PI concentrations. *n* = 3.

**Fig. 3:**
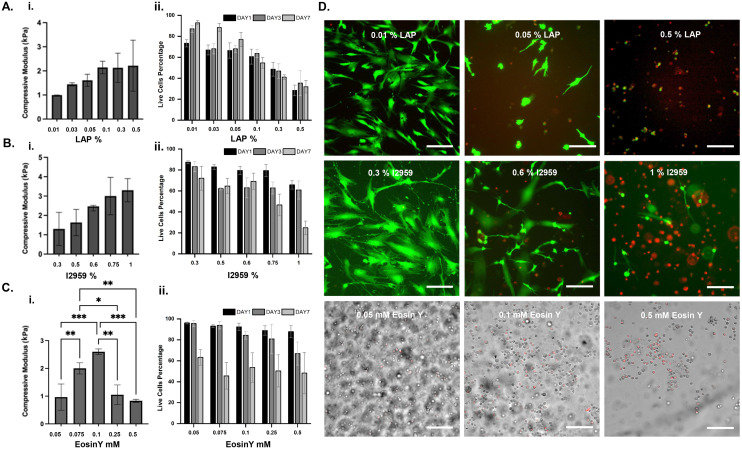
Characterization of A. LAP, B. I2959, C. Eosin Y in gelatin-based systems. i. Compressive modulus (kPa), ii. cell viability (%). D. Representative Live–Dead images for several concentrations of photo-initiators in GelMA 5% obtained at Day 7 after culture. For LAP and I2959, live cells are shown in green and dead cells are shown in red. For Eosin Y, the bright field images are shown in gray and dead cells are shown in red (see SI, Fig. S3–S5). Scale bars represent 100 μm. *n* = 3.

**Fig. 4:**
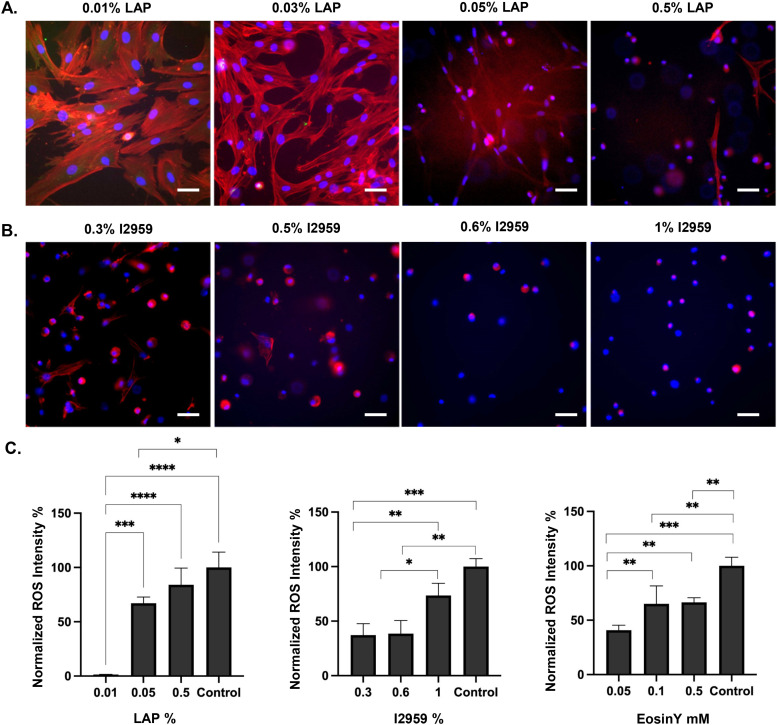
Focal adhesion staining images (red: actin, blue: DAPI) for A. LAP, B. I2959, in the gelatin-based system C, normalized intensities of ROS generation in hydrogels prepared with different concentrations of LAP, I2959, and Eosin Y. Scale bars represent 100 μm, *n* = 3.


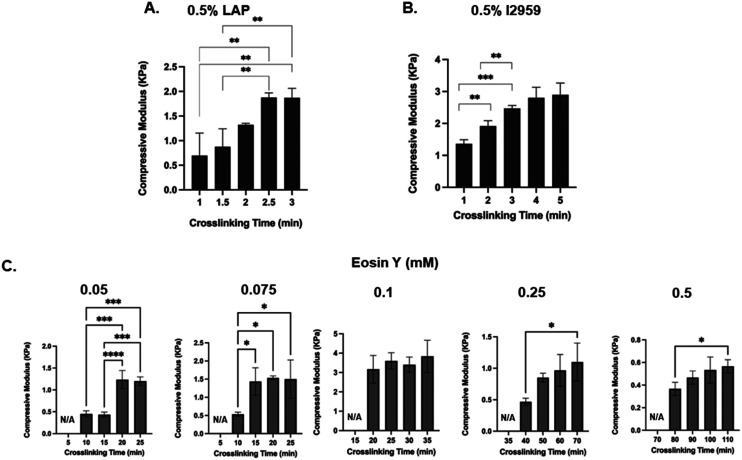
 Fig. S1. Crosslinking time optimization based on compressive modulus A. LAP, B. Irgacure 2959, C. Eosin Y in gelatin-based systems. μm. *n* = 3.


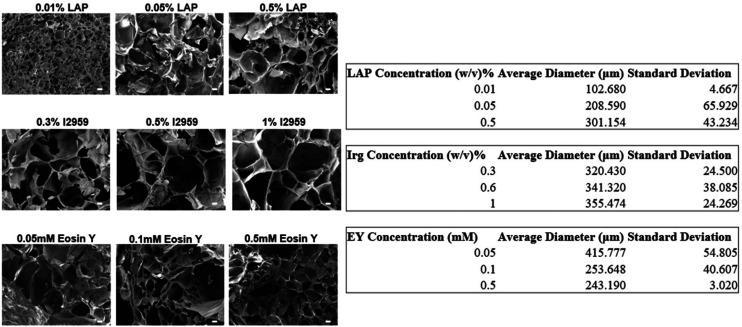
 Fig. S2. Representative SEM image of the obtained GelMA hydrogels and the analysis of the pore size variations obtained by ImageJ. Scale bar: 100 μm, *n* = 3.


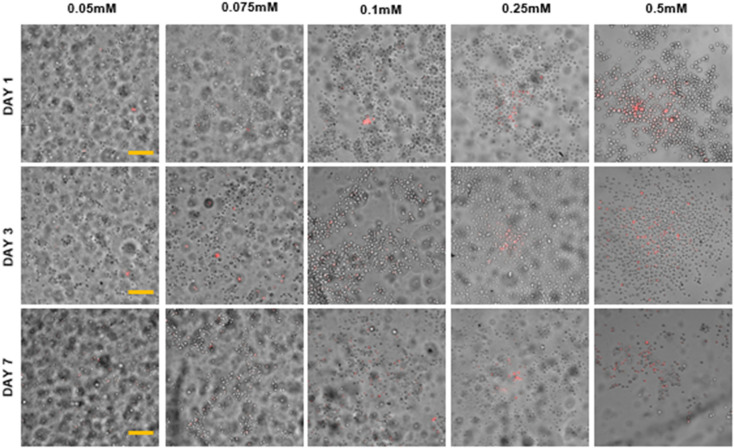
 Fig. S5. Live-dead images of MSCs in 5% GelMA with varying Eosin Y concentrations over 7 days. Red indicates dead cells; gray indicates visible cells with bright field.

The Royal Society of Chemistry apologises for these errors and any consequent inconvenience to authors and readers.

