# Supplementary material for: Design considerations for photoinitiator selection in cell-laden gelatin methacryloyl hydrogels
Source: Biomater Sci. 2025 Jul 15;14(3):807–16. doi: 10.1039/d5bm00550g (PMC12285922; doi:10.1039/d5bm00550g)
Supplement: BM-014-D5BM00550G-s001 [file BM-014-D5BM00550G-s001.pdf]

This journal is © The Royal Society of Chemistry 2025

Supplementary Materials

1.1 Determination of Optimal UV Crosslinking Time

To determine the optimal UV crosslinking time for LAP, I2959 and Eosin Y, modarate PI concentrations were selected in 5% GelMA hydrogels were tested across different crosslinking durations. Hydrogel samples (n=3) were prepared with 0.05% w/v LAP, 0.6% w/v I2959 and varying Eosin Y concentrations. Type I samples were crosslinked under 365 nm UV light at an intensity of 10 mW/cm^2 with crosslinking times ranging from 1 to 5 minutes. Type II samples were crosslinked for 5 to 110 min. The compressive modulus of the hydrogels and cell viability was assessed to ascertain the optimal crosslinking time for PIs. Threshold crosslinking times were determined for Eosin Y by increasing the crosslinking time gradually and conducting a compression test. For each group, crosslinking times were selected based on the first statistical significance observed in compressive modulus, indicating threshold crosslinking occurred (see **Fig. S1**). Compression experiments were conducted as indicated in the main text.

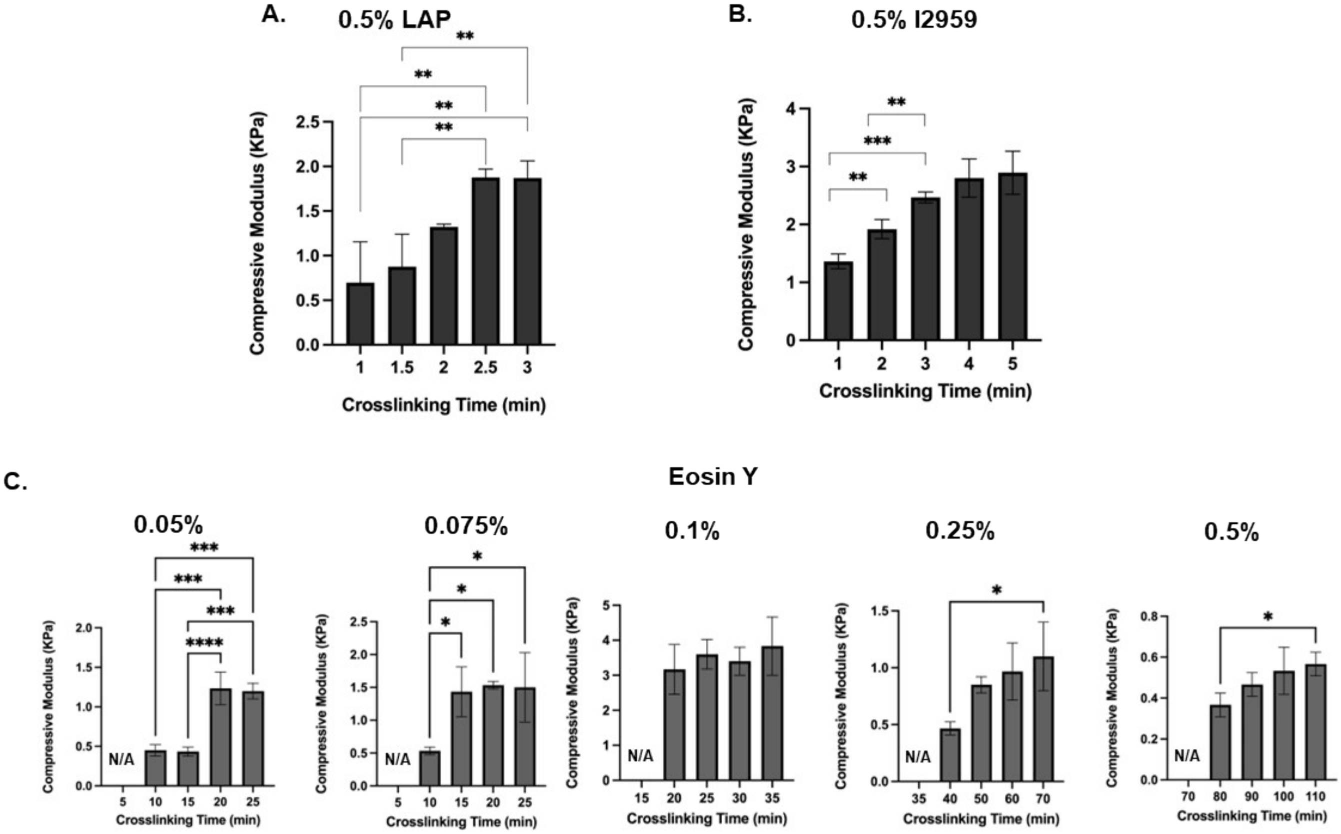

Figure S1. Crosslinking time optimization based on compressive modulus A. LAP, B. I2959, C. Eosin Y in gelatin-based systems. µm. n = 3.

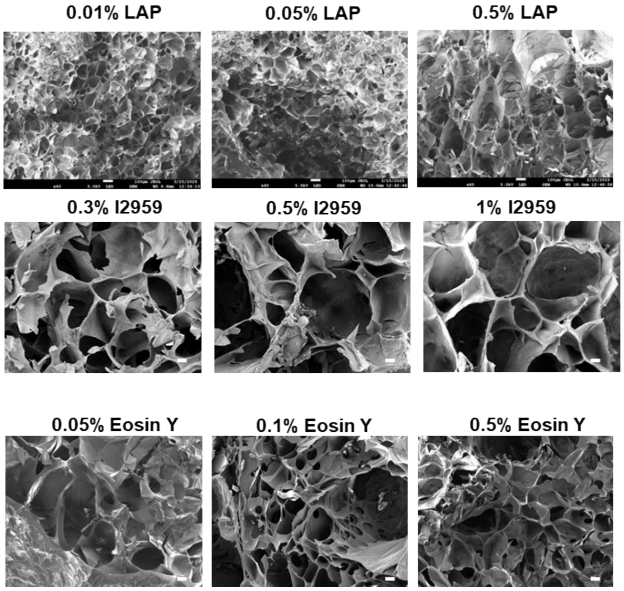

LAP -Diameter

LAP Concentration (w/v) %	Average Diameter (um)	Standard Deviation
0.01	102.6802	4.666904756
0.05	208.590375	65.92851496
0.5	301.15423	43.2335792

Irgacure -Diameter

Irg Concentration (w/v)%	Average Diameter (um)	Standard Deviation
0.3	320.43	24.49954
0.6	341.31965	38.0845591
1	355.473669	24.269432

Eosin Y -Diameter

EY Concentration (w/v) %	Average Diameter (um)	Standard Deviation
0.05	415.777313	54.8049298
0.1	253.647913	40.6074277
0.5	243.190042	3.01975843

Figure S2. Representative SEM image of the obtained GelMA hydrogels and the analysis of the pore size variations obtained by ImageJ. Scale bar: 100 µm, n = 3.

1.2 Live-Dead images

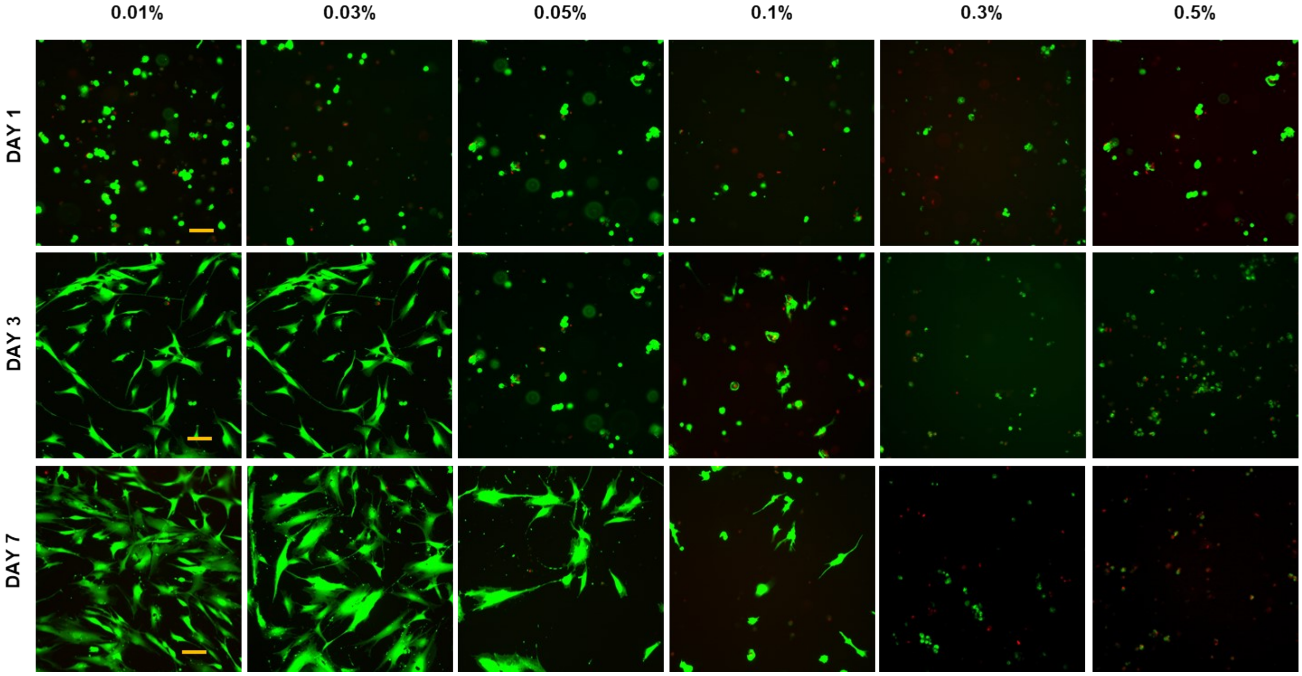

Figure S3. Live-dead images of MSCs in 5% GelMA with varying LAP concentrations over 7 days. Green indicates live cells; red indicates dead cells.

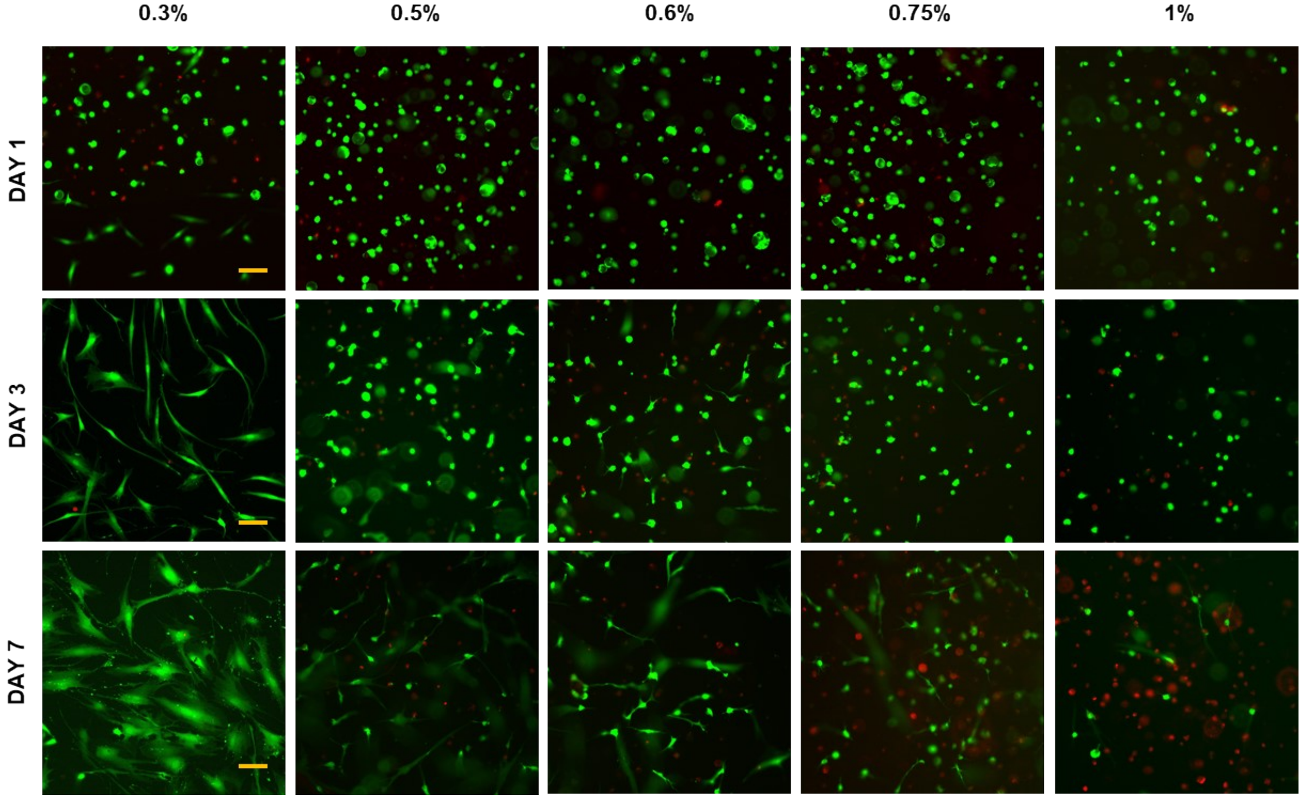

Figure S4. Live-dead images of MSCs in 5% GelMA with varying I2959 concentrations over 7 days. Green indicates live cells; red indicates dead cells.

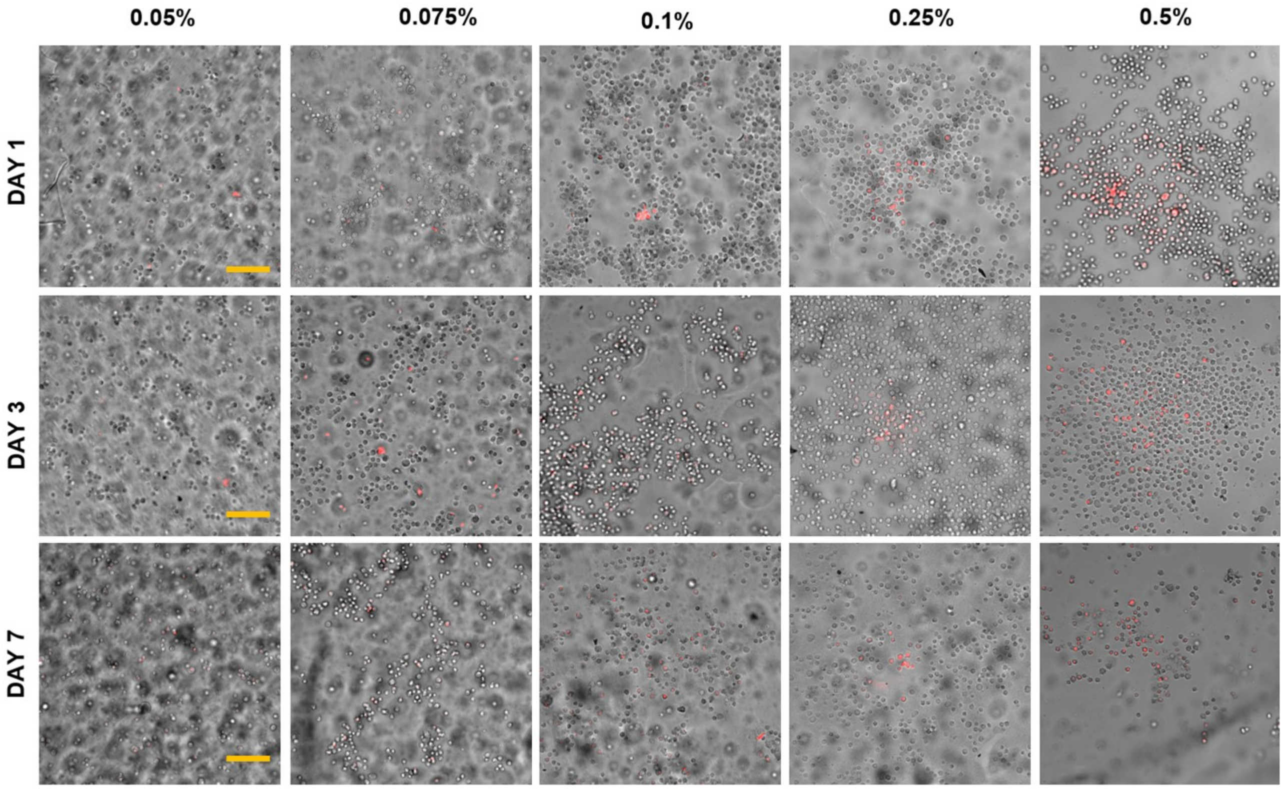

Figure S5. Live-dead images of MSCs in 5% GelMA with varying Eosin Y concentrations over 7 days. Red indicates dead cells; gray indicates visible cells with bright field.

1.3 Monitoring UV-vis profiles of PIs

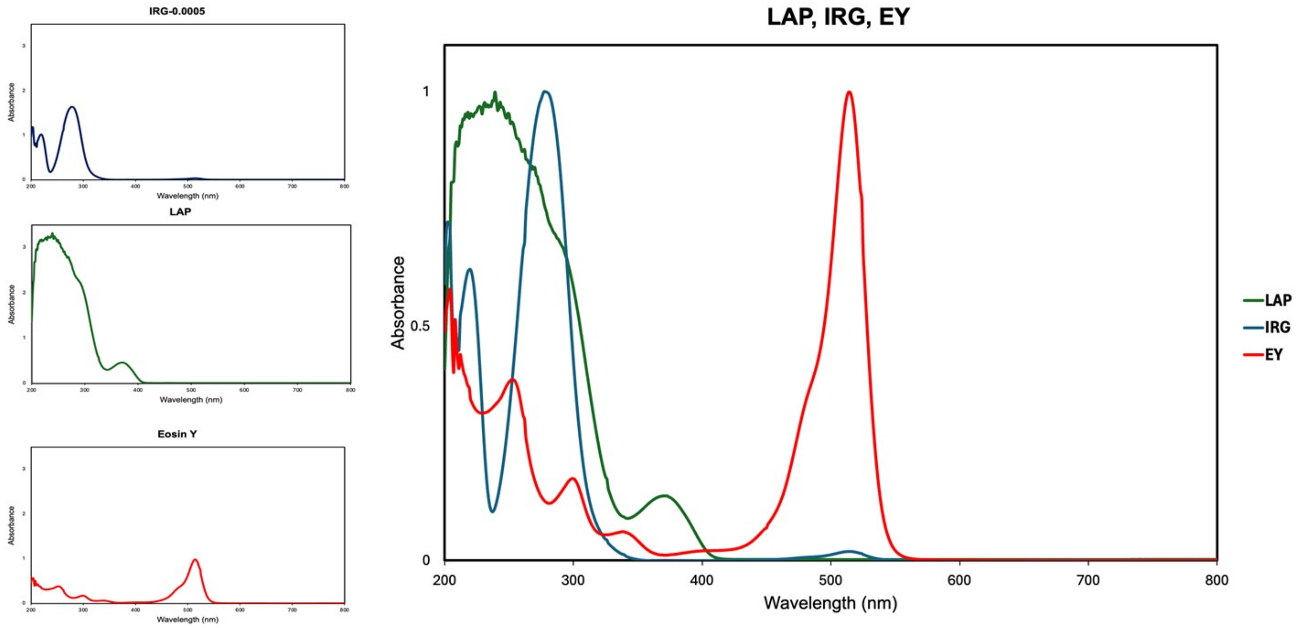

Figure S6. UV-vis profiles of LAP, I2959 and Eosin Y in DI water.

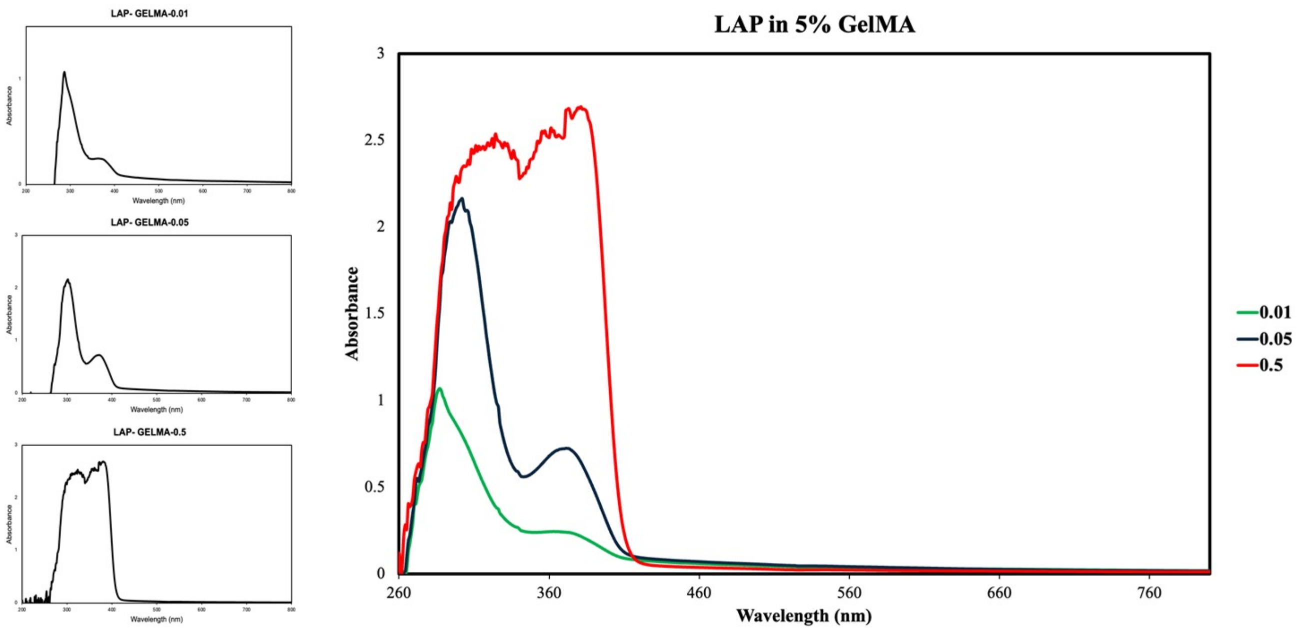

Figure S7. UV-vis profiles of varying concentration of LAP in 5% GelMA.

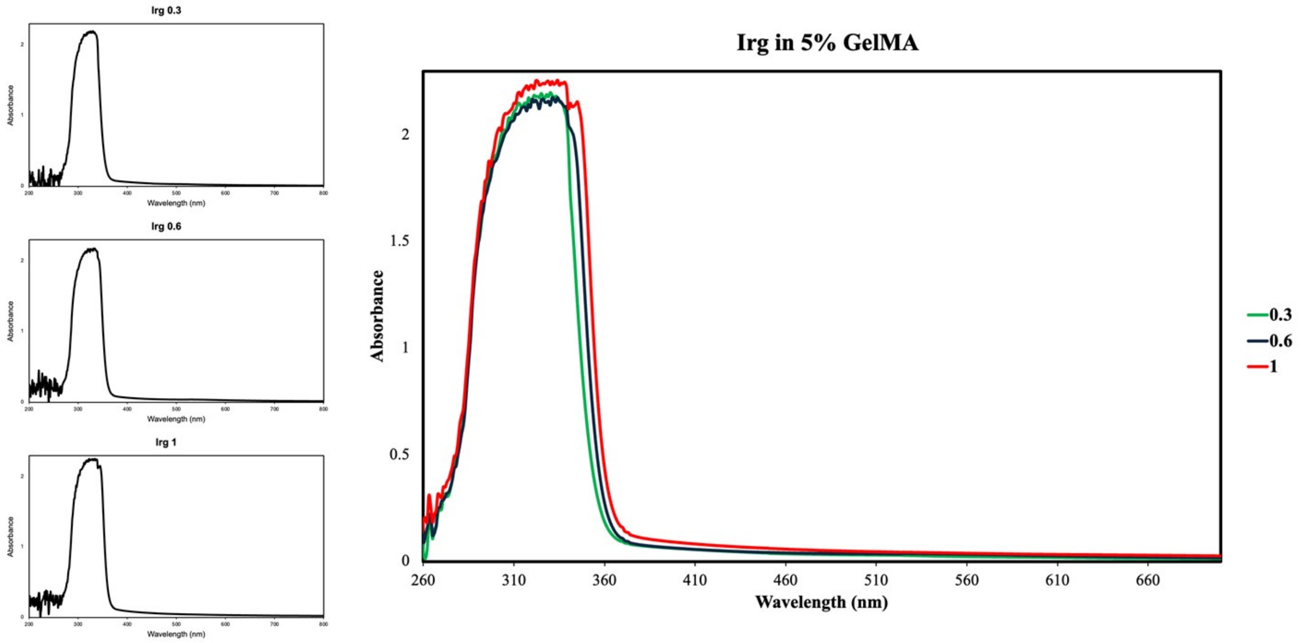

Figure S8. UV-vis profiles of varying concentrations of I2959 in 5% GelMA.

1.3 Monitoring PI degradation via UV-vis spectroscopy

The UV-Vis analysis provides insights into crosslinking density, PI efficiency, and PI consumption, which are crucial for optimizing hydrogel stability and performance in bioprinting. UV absorbance measurements of LAP, I2959, and Eosin Y were conducted using an UV-Visible double beam Spectrophotometer (UV-6300PC, VWR, Radnor, PA, USA) according to a well-

established protocol (32). Absorbance spectra for the PIs (at a concentration of 0.005% w/v) were recorded in both DPBS, and with varying concentrations of each PI in 5% GelMA. The spectra were obtained with background corrections using DPBS and GelMA at 37°C, respectively, in a UV-permissible cuvette (Spectrosil, VWR, Radnor, PA, USA) with a 1 cm path length. To calculate the decay constant, PIs were dissolved in DPBS (concentrations were optimized as 0.01% w/v for LAP and 0.05% w/v for I2959) and exposed to UV light (356 nm) in 10-second increments. Decay constants were determined through logarithmic transformation by linearizing the exponential data and obtaining the slope of the natural logarithm of absorbance over time. Absorbance was assumed to be directly proportional to concentration, as per Beer-Lambert's law. To model the degradation of the PI, we employed an exponential decay function:

$$C(t) = C_o e^{-I_o k.t^2} \tag{1}$$

where $C(t)$ is the molar concentration of the PI at time t, C_o is the initial molar concentration of PI and I_o is the incident light intensity. The decay parameter, k, represents the proportion of LAP and I2959 molecules cleaved per second:

$$k = \frac{\lambda}{hcN_A} \times \frac{f_A \phi}{C_i} \tag{2}$$

here λ is the wavelength of light, h is Planck's constant, c is the speed of light, N_A is Avogadro's constant, f_A represents the fraction of light absorbed by the PI (given by the Beer–Lambert law), ϕ is the quantum yield of photolysis (i.e. the fraction of absorbed photons that cleave a molecule), and C_i is the instantaneous molar concentration of PI molecules.

Note the first expression, when multiplied by I_o (as in eqn (1)) represents the flux of photons (in einsteins/cm^2). The fraction of light absorbed can be obtained from the Beer–Lambert law:

$$f_A = 1 - 10^{-\varepsilon C_i l} \tag{3}$$

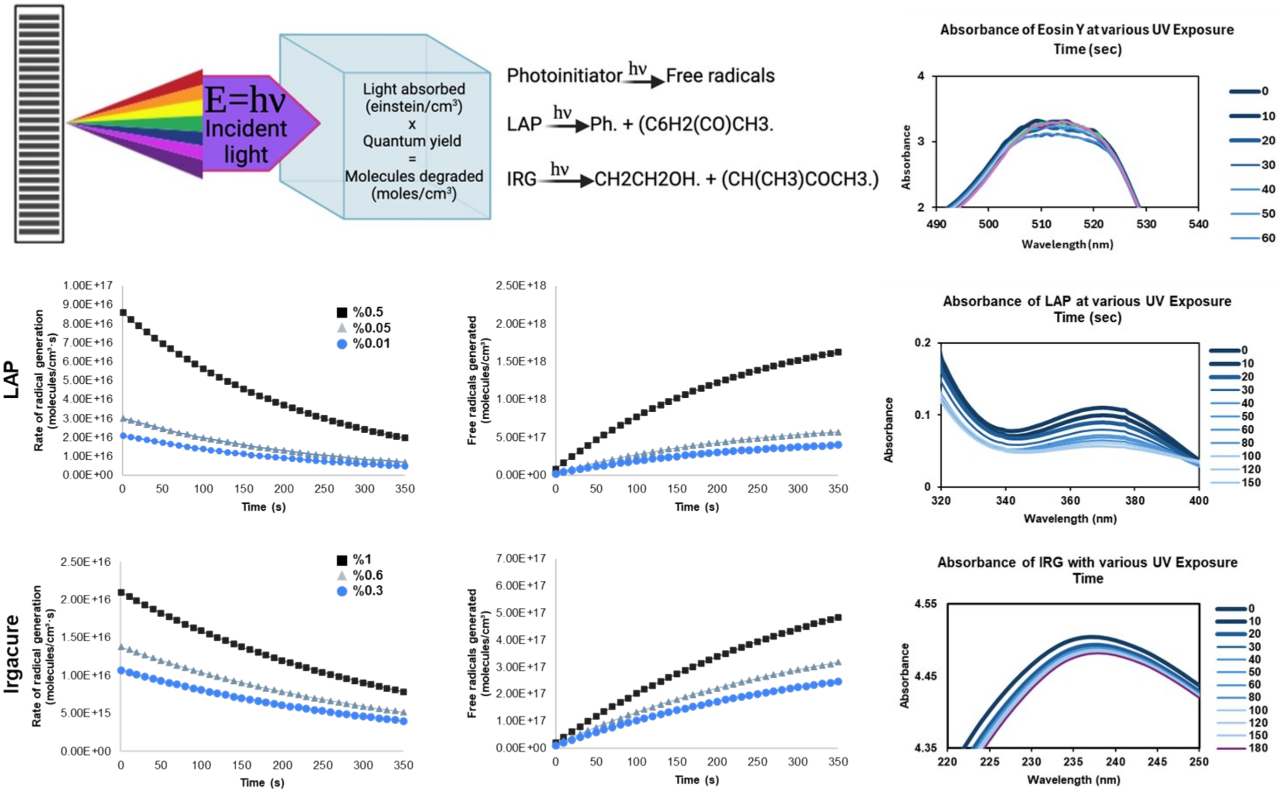

Figure S9. A) PI degradation is modelled for light exposed on a 1 cm cube (adapted by O'Connell et al.) B. UV-vis spectrographs of i. Eosin Y, ii. LAP, and iii. I2959 solution following sequential exposures to UV light to obtain decay parameters. C. The degradation model used to calculate free radical production across a range of PI concentrations. The right-hand side represents the rate of free radical production for LAP and I2959 in 5% GelMA for varying PI concentrations, while the left-hand side shows the total number of free radicals generated.
